# Cooperative MIMO Communication at Wireless Sensor Network: An Error Correcting Code Approach

**DOI:** 10.3390/s111009887

**Published:** 2011-10-20

**Authors:** Mohammad Rakibul Islam, Young Shin Han

**Affiliations:** 1 Electrical and Electronic Engineering Department, Islamic University of Technology, Boardbazar, Gazipur 1704, Dhaka, Bangladesh; E-Mail: rakibultowhid@yahoo.com; 2 School of Information and Communication Engineering, Sungkyunkwan University, Suwon 440-774, Korea

**Keywords:** cooperative technique, LDPC, BER, MIMO, wireless sensor networks

## Abstract

Cooperative communication in wireless sensor network (WSN) explores the energy efficient wireless communication schemes between multiple sensors and data gathering node (DGN) by exploiting multiple input multiple output (MIMO) and multiple input single output (MISO) configurations. In this paper, an energy efficient cooperative MIMO (C-MIMO) technique is proposed where low density parity check (LDPC) code is used as an error correcting code. The rate of LDPC code is varied by varying the length of message and parity bits. Simulation results show that the cooperative communication scheme outperforms SISO scheme in the presence of LDPC code. LDPC codes with different code rates are compared using bit error rate (BER) analysis. BER is also analyzed under different Nakagami fading scenario. Energy efficiencies are compared for different targeted probability of bit error *p_b_*. It is observed that C-MIMO performs more efficiently when the targeted *p_b_* is smaller. Also the lower encoding rate for LDPC code offers better error characteristics.

## 1. Introduction

Recent advances in micro-electro-mechanical systems technology have enabled the development of wireless sensor nodes in a wireless sensor network (WSN). These tiny sensor nodes are able to sense, process and communicate with each other [1,2]. Since the battery capacity in each node is limited and the goal is to maximize the lifetime of the network, there are strict energy consumption constraints in WSNs [3]. The size of sensors is typically small but the functions inside the sensor are complex. Recent hardware advancements allow more signal processing functionality to be integrated into a single sensor chip. RF transceiver, A/D and D/A converters, base band processors, and other application interfaces are integrated into a single device to be used as a smart wireless node. A wireless sensor network typically consists of a large number of sensor nodes distributed over a certain region. Monitoring node (MN) monitors its surrounding area, gathers application-specific information, and transmits the collected data to a data gathering node (DGN) or a gateway. Energy issues are more critical in the case of MNs rather than in the case of DGNs since MNs are remotely deployed and it is not easy to frequently change the energy sources. Therefore, the MNs have been the principal design issue for energy limited wireless sensor network design. One prospective solution is the use of MIMO [4,5] for energy efficient design with a targeted probability of bit error at the receiver. Also LDPC-coded MIMO optical communication is mentioned in [6]. But the MIMO techniques require complex transceiver circuitry and signal processing leading to large power consumptions at the circuit level. Moreover, physical implementation of multiple antennas at a small-size sensor node may not be feasible. The solution came in the form of cooperative MIMO (C-MIMO) [4–8]. C-MIMO is a kind of MIMO technique where the multiple inputs and outputs are formed via cooperation in a network of single antenna nodes. The sensors cooperate with each other to form a MIMO structure and in fact lead to better energy efficiency and smaller end-to-end delay. The basic idea of C-MIMO was first proposed by S. Cui in [4]. Later this idea has been improved in [5] by Jayaweera considering channel estimation (training overhead) in the DGN side and is further modified in [9] by Y. Gai and in [8] by M. Rakibul.

The issue of applying error control codes in WSNs is the topic of recent interest. The performance of block codes and Viterbi decoded convolutional codes is investigated in [10,11]. The iterative decoding algorithm using turbo code is used to prolong the network lifetime [12]. Low-density parity-check (LDPC) codes are more reliable than the block and convolutional codes and are serious competitors of turbo codes. In particular, LDPC codes exhibit an asymptotically better performance than turbo codes and admit a wide range of trade-offs between performance and decoding complexity [13]. Sartipi and Fekri [14] compare the performance of the LDPC codes and the Reed Solomon (RS) codes [15]. From the recent works, it is known that LDPC codes are attractive in WSNs because of their applications in compression, joint sourcechannel coding and distributed source coding [14,16]. However, to the best knowledge of the authors, there has been no document on the implementation of LDPC encoder/decoder in a wireless sensor node using cooperative communication. More precisely, none of the recent works have addressed the problem of reducing the energy consumption using error control coding. In this paper, LDPC code is incorporated in cooperative communication as an error control code. Later the idea is compared with SISO communication. In Section 2, the system model is shown and the error correction using LDPC code is analyzed in Section 3. Section 4 shows the energy model for both cooperative MIMO and SISO considering error correction codes. In Section 5, simulation results are shown and discussed. Finally, Section 6 concludes the paper.

## 2. Cooperative MIMO Communication

### 2.1. System Model

**Figure 1.**
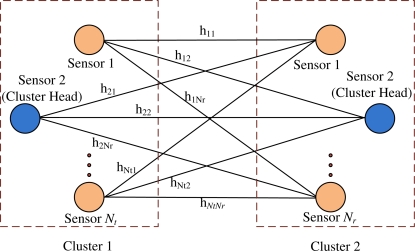
System model for cluster to cluster communication in wireless sensor network.

The system model for C-MIMO communication is a centralized wireless sensor network where there is a data gathering node (DGN) and several clusters with several sensors in each cluster. Sensors in one cluster transmit the data to the sensors in adjacent cluster and step by step the data reach the DGN. Figure 1 shows the cluster to cluster communication where two clusters are shown. The system considers *N_t_* number of sensors in the transmitting cluster, *N_r_* number of sensors in the receiving cluster and one antenna is placed at one sensor. Also, each element in the channel matrix *H* is assumed to be a zero-mean circularly symmetric complex Gaussian random variable with unit variance and can be considered as follows.

$$\text{H}=\left(\begin{array}{cccc} h_{11} & h_{12} & \ldots & h_{1N_{r}}\\ h_{21} & h_{22} & \ldots & h_{2N_{r}}\\ \vdots & \vdots & \vdots & \vdots \\ h_{N_{t}1} & h_{N_{t}2} & \ldots & h_{N_{t}N_{r}} \end{array}\right).$$

The problem here is stated from the receiver point of view, so a loss model is used to estimate the received energy. To calculate the total energy consumption, both the circuit and transmitter power are taken into count. The same transmitter and receiver blocks shown in [4] are used in this paper. Source coding, pulse shaping, and modulation block are as well omitted from the design. Throughout the paper, a system with narrowband, frequency-flat Rayleigh fading channels and perfectly synchronized transmission/reception between wireless sensor nodes is assumed. The fading is assumed constant during the transmission of each frame. In our model, a sensor with high residual energy is deployed as a cluster head and it remains the cluster head until the network dies. The cluster head broadcasts its status to the other sensors in the network. Each sensor node determines to which cluster it wants to belong by choosing the cluster head that requires the minimum communication energy. Once all the nodes are organized into clusters, each cluster head creates a schedule for the nodes in its cluster. This allows the radio components of each non-cluster-head node to be turned off at all times except during its transmit time, thus minimizing the energy dissipated in the individual sensors.

### 2.2. Cooperative Communication

**Figure 2.**
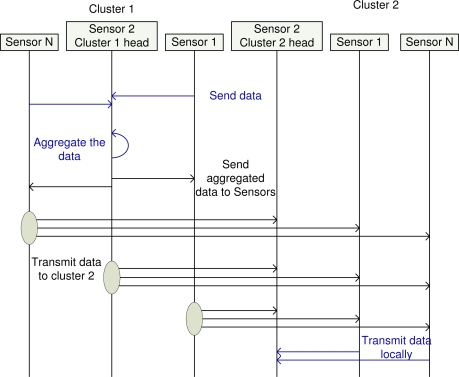
Cooperative communication.

The physical phenomena monitored by sensor networks, e.g., forest temperature, water contamination, usually yield sensed data that are strongly correlated. Data aggregation is the tool by which the correlated data size can be significantly reduced depending on the correlation factor. Figure 2 explains the cooperative communication where the sensors at cluster 1 send the information data to the cluster head os cluster 2. At the first step, the sensors at cluster 1 send the data to their cluster head. The cluster head then aggregates the data in the second step. After the aggregation, the cluster head send the aggregated data back to all the sensors in that cluster. This is the step three in cooperative communication. At this stage, all the sensors at cluster 1 have the same information data. At the fourth step, the sensors transmit the aggregated data to the cluster 2. After receiving the data at the receiving cluster, sensors at cluster 2 transmit the received data to their cluster head locally and complete the cooperative communication.

## 3. Error Correction Codes in Wireless Sensor Network

Error control coding (ECC) introduces redundancy into an information sequence *u* of length *k* by the addition of extra parity bits. Several different types of ECC exist, but we may loosely categorize them into two divisions: (1) block codes, which are of a fixed length *n_C_*, with *n_C_* − *k* parity bits, and are decoded into one block or codeword at a time; (2) convolutional codes, which, for a rate *k*/*n_C_* code, input *k* bits and output *n_C_* bits at each time interval, but are decoded in a continuous stream of length *L* >> *n_C_*. Block codes include repetition codes, Hamming codes [17], Reed Solomon codes, and BCH codes [18]. Short block codes like Hamming codes can be decoded by syndrome decoding or maximum likelihood (ML) decoding by either decoding to the nearest codeword or decoding on a trellis with the Viterbi algorithm or maximum a posteriori (MAP) decoding with the BCJR algorithm. Algebraic codes such as Reed Solomon and BCH codes are decoded with a complex polynomial solver to determine the error locations. Convolutional codes are decoded on a trellis using either Viterbi decoding, MAP decoding, or sequential decoding.

Another categorization is based on the decoding algorithms: (1) non-iterative decoding algorithms, such as syndrome decoding for block codes or maximum likelihood (ML) nearest codeword decoding for short block codes, algebraic decoding for Reed Solomon and BCH codes, and Viterbi decoding or sequential decoding for convolutional codes; (2) iterative decoding algorithms, such as turbo decoding with component MAP decoders for each component code, and the sum product algorithm (SPA) [19] or its lower complexity approximation, min-sum decoding [20], for low density parity check codes (LDPCs). The non-iterative decoding category may be further divided into hard and soft decision decoders; hard decision decoders output a final decision on the most likely codeword, while soft decision decoders provide soft information in the form of probabilities or log-likelihood ratios (LLRs) on the individual codeword bits. Viterbi decoding can be either hard decision or soft decision, with a 2*dB* gain in performance for soft decision decoding. Category (2) are all soft decision algorithms by nature, as iterative decoding requires soft information as *a priori* input for each iteration. Iterative decoding algorithms provide significant coding gain, at the cost of greater decoding complexity and power consumption. With the recent technological advancements, all these ECC techniques can be used in WSN. However, LDPC code is considered in this paper as an ECC tool at WSN for its superior error correcting capabilities.

### 3.1. Low Density Parity Check Codes

Low density parity check codes are codes specified by a matrix containing mostly 0’s and relatively few 1’s. A standard bipartite graph based ensemble which is shown in [21,22] is used in this paper. The code length is designated by *n* and number of constraints by *m*. Therefore, there are *n* variable nodes and *m* check nodes. Each variable node corresponds to one bit of the codeword and each check node corresponds to one parity check equation. Edges in the graph connect variable nodes to check nodes and represents the nonzero entries in *H* matrix. The term “low density” conveys the fact that the fraction of nonzero entries in *H* is small, in particular it is linear in the block length *n*, as compared to “random” linear codes for which the expected fraction of ones grows like *n*^2^ [23].

For regular codes, the corresponding *H* matrix has *δ_r_* ones in each row and *δ_c_* ones in each column. It means that every codeword bit participates in exactly *δ_c_* parity-check equations and that every such check equation involves exactly *δ_r_* codeword bits. Low density parity check codes have been constructed mostly using regular random bipartite graphs.

**Example 1**. Here is an example of a regular parity check matrix with *δ_r_* = 6 and *δ_c_* = 3.

$$H=\left[\begin{array}{cccccccccccc} 1 & 1 & 1 & 0 & 0 & 1 & 1 & 0 & 0 & 0 & 1 & 0\\ 1 & 1 & 1 & 1 & 1 & 0 & 0 & 0 & 0 & 0 & 0 & 1\\ 0 & 0 & 0 & 0 & 0 & 1 & 1 & 1 & 0 & 1 & 1 & 1\\ 1 & 0 & 0 & 1 & 0 & 0 & 0 & 1 & 1 & 1 & 0 & 1\\ 0 & 1 & 0 & 1 & 1 & 0 & 1 & 1 & 1 & 0 & 0 & 0\\ 0 & 0 & 1 & 0 & 1 & 1 & 0 & 0 & 1 & 1 & 1 & 0 \end{array}\right]$$

**Figure 3.**
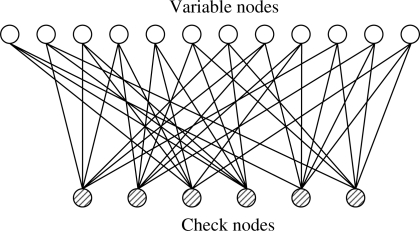
Bipartite graph corresponding to a regular parity check matrix.

The bipartite graph corresponding to this parity check matrix is shown in Figure 3.

For irregular codes, *δ_r_* and *δ_c_* are not fixed for every row and column of the parity check matrix. We consider that the irregular bipartite graph has a maximum variable side degree *δ_r_* and a maximum check side degree *δ_c_*.

**Example 2**. The following *H* matrix is an example of an irregular parity check matrix with a maximum *δ_r_* = 6 and a maximum *δ_c_* = 3.

$$H=\left[\begin{array}{cccccccccccc} 1 & 0 & 1 & 0 & 0 & 1 & 1 & 0 & 0 & 0 & 1 & 0\\ 1 & 1 & 0 & 1 & 1 & 0 & 0 & 0 & 0 & 0 & 0 & 1\\ 0 & 0 & 0 & 0 & 0 & 1 & 1 & 1 & 0 & 1 & 1 & 1\\ 1 & 0 & 0 & 1 & 0 & 0 & 0 & 1 & 1 & 1 & 0 & 1\\ 0 & 1 & 0 & 1 & 1 & 0 & 1 & 1 & 1 & 0 & 0 & 0\\ 0 & 0 & 1 & 0 & 1 & 1 & 0 & 0 & 0 & 1 & 0 & 0 \end{array}\right]$$

**Figure 4.**
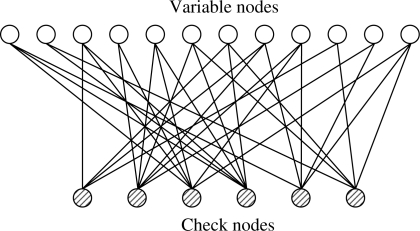
Bipartite graph corresponding to an irregular parity check matrix.

The bipartite graph corresponding to this parity check matrix is shown in Figure 4.

**Figure 5.**
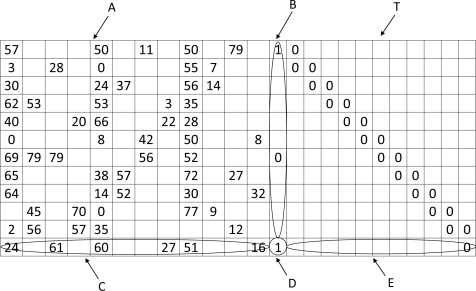
*H* matrix used in the simulation.

For this paper, H matrix shown in Figure 5 is used for simulation. This H matrix is a special matrix used in 802.11n standard. WSN is energy constraint in nature and the sensors work as intermediate devices when the data are transferred from a designated area to the data gathering node (DGN). Since decoding can be performed in the DGN, energy efficient decoding technique is not a concern for this paper. Encoding is one critical issue considered in the wireless sensor network. In this paper, Richardson encoding scheme is used as a tool for using LDPC code in WSN and is explained in the next subsection.

### 3.2. Richardson Scheme as the Encoding Technique

The encoding method proposed by Richardson *et al.* [13] assumes that *H* can be converted to an approximate lower triangular matrix. The authors worked on an *m* × *n* parity check matrix *H* over *F* where *n* is the number of variable nodes and *m* is the number of check nodes. The parity check matrix *H* is transformed in the form of

(1) $$H=\left[\begin{array}{ccc} A & B & T\\ C & D & E \end{array}\right],$$

where *A* is (*m* − *g*) × (*n* − *m*), *B* is (*m* − *g*) × *g*, *T* is (*m* − *g*) × (*m* − *g*), *C* is *g* × (*n* − *m*), D is *g* × *g*, and E is *g* × (*m* − *g*) where *g* is denoted as the gap. Further, all these matrices are sparse and *T* is lower triangular with ones along the diagonal. This matrix is multiplied from the left by

(2) $$\left[\begin{array}{cc} I & 0\\ -ET^{-1} & I \end{array}\right]$$

And the *H* matrix is found as

(3) $$\left[\begin{array}{ccc} A & A & T\\ -ET^{-1}A+C & -ET^{-1}B+D & 0 \end{array}\right].$$

They then break the codeword as *x* = (*s*, *p*_1_, *p*_2_) where *s* denotes the systematic part, *p*_1_ and *p*_2_ denote the parity part, *p*_1_ has length *g*, and *p*_2_ has length (*m* − *g*). After that, the equation *Hx^T^* = 0*^T^* is used to state the following two equations

(4) $$As^{T}+{\mathrm{Bp}}_{1}^{T}+{\mathrm{Tp}}_{2}^{T}=0,$$

(5) $$(-{\mathrm{ET}}^{-1}A+C)s^{T}+(-{\mathrm{ET}}^{-1}B+D)p_{1}^{T}=0.$$

Taking (−*ET*^−1^*B* + *D*) as nonsingular, it is concluded that

(6) $$p_{1}^{T}=-{\left(-{\mathrm{ET}}^{-1}B+D\right)}^{-1}(-{\mathrm{ET}}^{-1}A+C)s^{T}.$$

(7) $$p_{2}^{T}=-T^{-1}({\mathrm{As}}^{T}+{\mathrm{Bp}}_{1}^{T}).$$

By using the step by step procedure, it is shown that the complexity of calculating *p*_1_ and *p*_2_ are *O*(*n* + *g*^2^) and *O*(*n*) respectively. The matrix used in our simulation can also be written in ALT form and is shown in Figure 5.

## 4. Energy Model for Cooperative Communication Using LDPC Code

The total power consumption *P_T_* for a single node consists of two main parts, namely, the power consumption of all the power amplifiers *P_PA_* which is a function of transmission power *P_out_*, and the power consumption of all other circuit blocks *P_C_*. Thus one can write

(8) $$P_{T}=P_{\mathrm{PA}}+P_{C}.$$

The power consumption of all the power amplifiers can be calculated using the following equation

(9) $$P_{\mathrm{PA}}=(1+\alpha )P_{\mathrm{out}},$$

where $\alpha =(\frac{\xi }{\eta }-1)$, where *η* is the drain efficiency [24] and *ξ* is the peak to average ratio. When the channel only experiences a *k^th^* power path loss with additive white Gaussian noise (AWGN), *P_out_* can be calculated using the link budget relationship as follows.

(10) $$P_{\mathrm{out}}={\bar{E}}_{b}R_{b}\times \frac{{\left(4\pi \right)}^{2}d^{k}}{G_{t}G_{r}\lambda^{2}}M_{l}N_{f},$$

where *Ē_b_* is the average energy per bit required for a given bit error rate (BER) specification, *R_b_* is the transmission bit rate, *d* is the transmission distance, *G_t_* and *G_r_* are the transmitter and receiver antenna gains respectively, *λ* is the carrier wavelength, *M_l_* is the link margin compensating the hardware process variations and other background noise, *N_f_* is the receiver noise figure defined as $N_{f}=\frac{N_{r}}{N_{0}}$ where *N_r_* is the power spectral density (PSD) of the total effective noise at the receiver input and *N*_0_ is the single-sided thermal noise PSD at the room temperature.

The power consumption in the circuit block includes transmitter and receiver power consumption *P_ct_* and *P_cr_*, respectively. This power consumption is due to several power blocks such as *P_mix_*, *P_syn_*, *P_filt_*, *P_filr_*, *P_LNA_*, *P_IFA_*, *P_DAC_*, and *P_ADC_* which are the power consumption values of the mixer, the frequency synthesizer, the active filters at the transmitter and at the receiver side, the low noise amplifier, the intermediate frequency amplifier, the D/A and A/D converter, respectively. The power consumption block for error correction is not considered as it is same for cooperative case and SISO case. The total energy consumption per bit can be written as

(11) $$E_{\mathrm{bt}}\frac{\left(P_{\mathrm{PA}}+P_{C}\right)}{R_{b}},$$

where *R_b_* is the actual bit rate and can be replaced by $R_{b}^{\mathrm{eff}}=\frac{F-{\mathrm{pN}}_{T}}{F}R_{b}$ when *pN_T_* training symbols are inserted in each block to estimate the channel at the receiving cluster or DGN side. The block size is equal to *F* symbols and can be obtained by setting *F* = ⌊*T_C_R_S_*⌋, where *R_S_* is the symbol rate and *T_C_* is the fading coherence time. The fading coherence time can be estimated from $T_{C}=\frac{3}{4f_{m}\sqrt{\pi }}$ where the maximum doppler shift *f_m_* is given by $f_{m}=\frac{v}{\lambda }$ with *v* being the velocity and λ being the carrier wavelength [25]. The total energy consumption is estimated by multiplying *E_bt_* by the number of bits *L* to be transmitted. Now we develop the mathematical model where we estimate total energy consumption for cooperative communication.

The total energy consumption in cooperative case is modeled as

(12) $$\begin{array}{lll} E_{\mathrm{CO}} & = & \sum_{i=1}^{N_{t}-1}L_{i}E_{i}^{t}+E_{\mathrm{da}}\sum_{i=1}^{N_{t}}L_{i}+E_{\mathrm{enc}}\sum_{i=1}^{N_{t}}\frac{L_{i}}{r}\gamma_{i}\\ & & +(N_{t}-1)E_{i}^{t0}\sum_{i=1}^{N_{t}}\frac{L_{i}}{r}\gamma_{i}\\ & & +E_{M}^{l}\sum_{i=1}^{N_{t}}\frac{L_{i}}{r}\gamma_{i}\\ & & +\frac{1}{b_{\mathrm{mimo}}}\sum_{i=1}^{N_{t}}\sum_{i=1}^{N_{r}}\frac{L_{i}}{r}\gamma_{i}b_{l_{r}}E_{j}^{t}, \end{array}$$

The energy per bit $E_{i}^{t}$ is needed to transmit the data from sensors to the cluster head. *E_da_* is the energy dissipation per bit required in the cluster head for data aggregation. It depends on the algorithm complexity and can be expressed as

(13) $$E_{\mathrm{da}}(L)=\{\begin{array}{ll} C_{0}+C_{1}\times L+C_{2}\times L^{2} & \text{for}\;O(n^{2})\\ C_{0}+C_{1}\times L & \text{for}\;O(n) \end{array},$$

where L is the number of transmission bits and *C*_0_, *C*_1_ and *C*_2_ are coefficients depending on the software and CPU parameters. *E_enc_* is the encoding energy per bit and is taken 1 *μJ* [26]. $E_{i}^{t0}$ denotes the local transmission energy cost per bit for transferring the aggregated data to the remaining active sensors, *γ* is the percentage of remaining data after aggregation and it reflects the correlation between data amongst different sensors. *r* is the rate of LDPC encoding. Since the use of a rate *r* = 1/2 makes the size of the data after encoding, 2 times the original data size, the $\frac{L_{i}}{r}$ term is used to represent the data size after encoding a message size of *L_i_* with rate *r*. The same energy per bit $E_{i}^{t0}$ is needed to transmit a command signal from the cluster head to the selected sensors. After receiving all the bits, the nodes encode the transmission sequence according to some diversity scheme, such as the STBC. $E_{M}^{l}$ denotes the energy cost per bit for the long haul MIMO transmission [4]. $\sum_{i=1}^{N_{t}}\frac{L_{i}}{r}\gamma_{i}$ is divided by the optimal bit size of the long haul transmission *b_mimo_* to find the number of symbols present in the received signal. The number of symbols is then multiplied by the optimal bit size of the local transmission *b_l_r__* to find the total bit length. $E_{j}^{t}$ is the energy per bit required to transmit the data from a sensor to the cluster head at the receiver side. *N_r_* is the number of sensors at the receiving cluster.

For the SISO approach, sensors transmit their data to the cluster head and as there is no burden for channel estimation, the cluster head will transmit all the aggregated data directly to the destination node without any cooperation. So the total energy consumption becomes

(14) $$E_{\mathrm{SISO}}=\sum_{i=1}^{N_{t}-1}L_{i}E_{i}^{t}+E_{\mathrm{da}}\sum_{i=1}^{N_{t}}L_{i}+E_{\mathrm{enc}}\sum_{i=1}^{N_{t}}\frac{L_{i}}{r}\gamma_{i}+E_{S}^{l}\sum_{i=1}^{N_{t}}\frac{L_{i}}{r}\gamma_{i},$$

where $E_{S}^{l}$ denotes the SISO long haul transmission and can be calculated as a special case of MIMO transmission with *N_t_* = 1 and *N_r_* = 1. In both SISO and C-MIMO case, fixed constellation size is used. Since the encoding energy using Richardson scheme is same for both C-MIMO and SISO approach, it is not considered in the equation for C-MIMO and SISO.

## 5. Simulation Results and Discussion

In order to get the total communication energy consumption, the average energy per bit required for a given BER *P_b_*, *Ē_b_* needs to be determined. In this approach, the value of *Ē_b_* is found by using a numerical search. Ten thousand randomly generated channel samples are taken and averaged to find the desired bit error rate at each transmission distance. The value of the constellation size is kept fixed. For the long haul communication, SISO is taken as a special case of MIMO structure. A list of system parameters used in our simulation is shown in Table 1 where the power consumption values of various circuit blocks are quoted from [4].

### 5.1. Energy Issue

**Figure 6.**
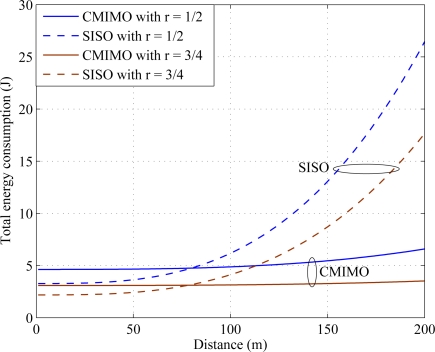
Total energy consumption over distance.

Total energy consumption and energy efficiency are the key terms to evaluate the energy efficient performance. For simulation, it is considered that all the sensors in a cluster are transmitting the same data size of *L_i_* = 10 *kb*. The simulation is performed based on the cluster size of *N_t_* = 4. In Figure 6, the total energy consumption over distance is shown for cluster to cluster data transmission. From Figure 6 it is clear that the cooperative MIMO is more energy efficient than SISO transmission. The simulation is taken for two different code rates *r* = 1/2 and *r* = 3/4. When the code rate increases, parity bits compared to message bits are reduced. Therefore, total energy consumption reduces. This is verified in Figure 6.

### 5.2. Delay Issue

The total delay required is defined as the total transmission delay. For a fixed transmission bandwidth *B*, we assume that the symbol period is approximately *T_S_* ≈ 1/*B*. The total delays in the case of SISO communication is defined as

(15) $$T_{\mathrm{SISO}}=T_{S}\left(\sum_{i=1}^{N_{t}}\frac{L_{i}}{b_{l_{t}}}+\frac{1}{b_{\mathrm{SISO}}}\sum_{i=1}^{N_{t}}\frac{L_{i}}{r}\gamma_{i}\right)+t_{\mathrm{da}},$$

Where *b_l_t__* is the transmission bit size at the transmitter side local communication and *b_SISO_* is the transmission bit size for long haul SISO transmission. *t_da_* is the time taken for data aggregation.

The total delays in the case of cooperative MIMO communication is defined as

(16) $$\begin{array}{lll} T_{\mathrm{CO}} & = & T_{S}\left(\sum_{i=1}^{N_{t}}\frac{L_{i}}{b_{l_{t}}}+\sum_{i=1}^{N_{t}}\frac{\frac{L_{i}}{r}\gamma_{i}}{b_{l_{t}}}+\frac{1}{b_{\mathrm{MIMO}}}\sum_{i=1}^{N_{t}}\frac{L_{i}}{r}\gamma_{i}\right)\\ & & +T_{S}\frac{1}{b_{l_{r}}}\left\{\sum_{i=1}^{N_{t}}\frac{L_{i}}{r}\gamma_{i}\right\}+t_{\mathrm{da}}+t_{\mathrm{ch}}, \end{array}$$

Where *t_ch_* and *t_da_* are the channel estimation and data aggregation delays respectively. The term $T_{S}\sum_{i=1}^{N_{t}}\frac{L_{i}}{b_{l_{t}}}$ is for the delay due to the local transmission from sensors to the cluster head. The next term is due to the local transmission from cluster head to the sensors. $T_{S}\frac{1}{b_{\mathrm{MIMO}}}\sum_{i=1}^{N_{t}}\frac{L_{i}}{r}$ term is caused by the long haul MIMO transmission. The next term is due to the local transmission at the receiver side. The assisting nodes first quantize each symbol they receive into *n_r_* bits, then transform all the bits into symbols using *b_l_r__* and transmit to the cluster head to do the joint detection.

The delay difference is calculated using the following equation. We assume the value of *t_ch_* ≈ 0.

(17) $$\begin{array}{lll} \mathrm{DD} & = & T_{\mathrm{SISO}}-T_{\mathrm{CO}}\\ & = & T_{S}\left(\frac{1}{b_{\mathrm{SISO}}}\sum_{i=1}^{N_{t}}\frac{L_{i}}{r}\gamma_{i}-\sum_{i=1}^{N_{t}}\frac{\frac{L_{i}}{r}\gamma_{i}}{b_{l_{t}}}\right)\\ & & -T_{S}\frac{1}{b_{\mathrm{MIMO}}}\sum_{i=1}^{N_{t}}\frac{L_{i}}{r}\gamma_{i}-T_{S}\frac{1}{b_{l_{r}}}\sum_{i=1}^{N_{t}}\frac{L_{i}}{r}\gamma_{i}, \end{array}$$

**Figure 7.**
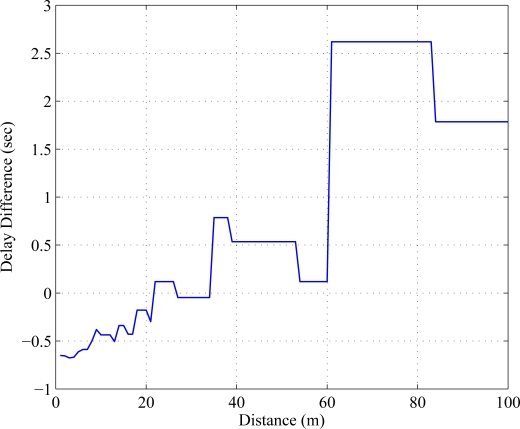
Delay difference over distance for code rate $r=\frac{3}{4}$.

The value of *n_r_* is chosen at the receiver based on the optimized transmitted constellation size. The delay difference is a measure of delay performance by which the cooperative MIMO can be compared with SISO. Positive delay difference indicates the SISO is facing larger delay compared to C-MIMO. In Figure 7, delay difference is compared where proposed C-MIMO outperforms SISO after 60 meters.

### 5.3. Constellation Size Issue

**Figure 8.**
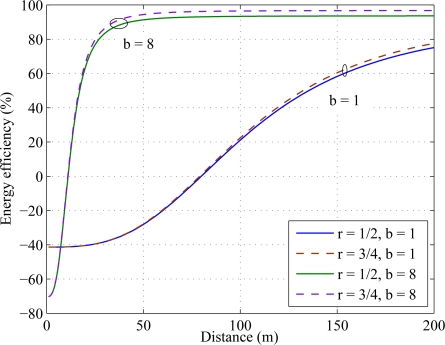
Energy efficiency for different constellation sizes.

Since Energy efficiency = {*E_SISO_* − *E_C−MIMO_*}/*E_SISO_*, positive energy efficiency indicates {*E_SISO_* > *E_C−MIMO_*}. In Figure 8, energy efficiency is simulated over distance for different constellation sizes. The simulation results show that for rate *r* = 1/2, cooperative MIMO outperforms SISO after 80 meters for constellation size *b* = 1 whereas it takes 10 meters for constellation size *b* = 8.

### 5.4. Bit Error Rate Issue

**Figure 9.**
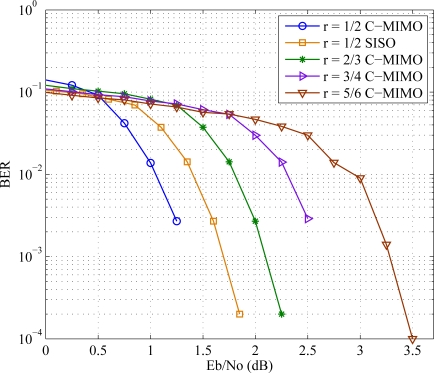
Bit error rate over SNR curve for different encoding rate.

Using the parity check matrix provided in IEEE 802.11*n* standard shown in Figure 5, comparative error performance studies have been taken for different encoding rates and are shown in Figure 9. Also the C-MIMO is compared with SISO in the $\frac{1}{2}$ rate case. The codeword length is kept fixed and the number of decoder iteration is taken as 100. Bit error rate (BER) is taken as performance parameter in this paper. BPSK modulation and AWGN channel are used for the simulation. Like the other wireless channels, simulation using cooperative MIMO shows similar outcomes.

**Figure 10.**
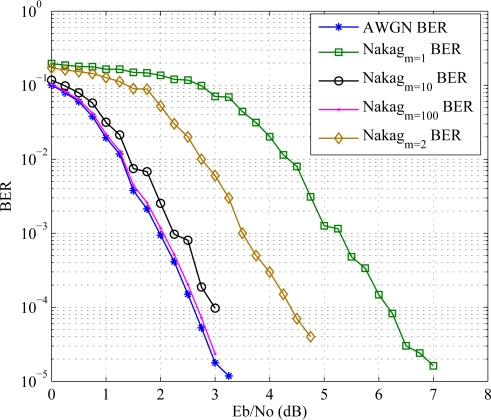
Error performance for different Nakagami coefficient *m* in Nakagami fading channel.

The same BER analysis is taken in Figure 10 in a Nakagami fading channel scenario. The result shows that the decrease in Nakagami coefficient *m* degrades the error performance.

### 5.5. Reception Quality Issue

**Figure 11.**
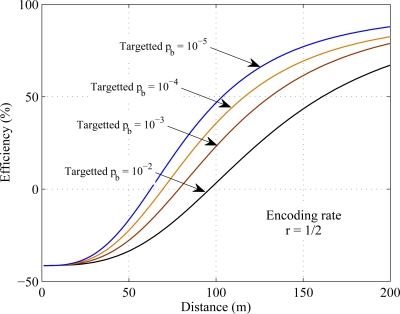
Energy efficiency for different targeted probability of bit error *p_b_*.

Targeted BER is the parameter that indicates the reception quality of the signal. The cooperative MIMO communication used in this paper is simulated with a fixed value of targeted BER. Figure 11 shows that the change in targeted BER changes the efficiency of cooperative communication over SISO transmission. Result shows that the cooperative communication is more energy efficient than SISO transmission in smaller targeted BER.

## 6. Conclusions

Energy efficient data transmission is one of the key factors for energy constraint wireless sensor network. An energy efficient cooperative technique considering low density parity check codes is modeled and simulated using Matlab. The results show that the cooperative communication outperforms SISO transmission at the presence of error correction code. The energy efficiency remains almost unchanged in different encoding rates but it largely varies with the change in constellation size. BER analysis is also taken to show the similar error characteristics in the cooperative MIMO environment. Data with smaller encoding rate shows better BER results than larger encoding rate for a fixed SNR. Simulation is also performed in the situation of a fading environment. It is also found that cooperative communication is more energy efficient than SISO transmission in smaller targeted BER. Therefore it can be concluded that cooperative MIMO with LDPC can be a good choice for high reception quality signals.

## Figures and Tables

**Table 1.** System Parameters.

*f_c_* = 2.5 GHz	*η* = 0.35
*G_t_G_r_* = 5 dBi	*N*_0_ = −171 dBm/Hz
*B* = 10 KHz	k = 2 for local com
*P_b_*= 10^−3^	k = 3 for long haul com
*N_f_* = 10 dB	*M_l_* = 40 dB
*P_syn_* = 50.0 mW	*P_mix_* = 30.3 mW
*E_da_* = 5 nJ/bit/signals	*P_LNA_* = 20 mW
*P_fil_t__* = 2.5 mW	*P_fil_r__* = 2.5 mW
